# Influence of Different Parameters on the Performance of Alkali-Activated Slag/Fly Ash Composite System

**DOI:** 10.3390/ma15082714

**Published:** 2022-04-07

**Authors:** Zhipeng Zhang, Yanmin Jia, Jinliang Liu

**Affiliations:** School of Civil Engineering, Northeast Forestry University, Harbin 150040, China; zhipeng2019@nefu.edu.cn (Z.Z.); jinliangliu@nefu.edu.cn (J.L.)

**Keywords:** alkali-activated, fly ash, slag, orthogonal test

## Abstract

In order to study the influence law of each parameter on the performance of the alkali-activated composite gelling system, the influence degree was sorted, and the most important parameter affecting each performance was found. The solution of liquid water glass and solid sodium hydroxide was used as the alkaline activator, and the mixing ratio was designed by the orthogonal test method. The effects of four parameters of fly ash content, water glass modulus, water glass solid content, and water–solid ratio on the working performance and mechanical properties of alkali-activated slag–fly ash composite cementation system were discussed. The gelling system was studied by microscopic experiments such as SEM and FTIR. The results show that the solid content of water glass has the greatest influence on the fluidity of the composite cementitious system, and the content of fly ash is the primary factor affecting the setting time of the material. The flexural and compressive strengths at the age of 7 d and 28 d were most affected by the content of fly ash, and the solid content of water glass had the greatest influence on the flexural and compressive strengths at the age of 2 d. From the perspective of microscopic morphology, in the high-strength samples, the fly ash particles and the remaining outer shell are embedded in the gel to form a dense whole. When the amount of silica in the composite gelling system is too high, it will cause the phenomenon of low macroscopic mechanical properties.

## 1. Introduction

Cement occupies a vital position in modern civilization, with global cement production averaging more than half a ton per person per year. However, while cement promotes the development of human society, it also brings serious environmental problems. The cement industry is an intensive source of fuel consumption and greenhouse gas emissions. The annual production of cement worldwide accounts for about 7% of global CO_2_ emissions, and it is one of the main industrial sources of carbon dioxide emissions [1,2]. In 1990, the global cement industry only emitted 576 million tons of carbon dioxide, and in 2014 it reached 2.083 billion tons, a more than three-fold increase in emissions. If the current rate of CO_2_ emissions continues and no further emission reduction strategies are implemented, it is estimated that by 2050, the global cement industry’s CO_2_ emissions will reach 2.34 billion tons [3]. As cement production continues to increase, the industry is under pressure to reduce energy use and CO_2_ emissions, and there is an urgent need for new cementitious materials as alternatives to cement that reduce environmental impact and improve sustainability [4,5,6,7]. Among them, the alkali-activated material is a new type of inorganic aluminosilicate cementitious material, which is considered to be an ideal substitute for ordinary Portland cement. Its preparation process has low energy consumption and greatly reduces carbon dioxide emissions [8,9,10,11], has the advantages of ordinary Portland cement, and has good properties (high early strength, good acid and sulfate resistance, etc.) [4,12,13,14,15]. Common alkali-activated raw materials are ground blast furnace slag, fly ash, and kaolin. The alkali activity of these three materials is slag, kaolin, and fly ash in descending order [16,17,18]. The slag alkali excitation reaction is fast and the strength is stable, but its working performance is relatively poor. Slag and fly ash hybrid systems are the most popular, they are low-cost industrial by-products and are the subject of this study [19].

For alkali-activated materials, relevant scholars have studied the influence of factors such as the amount of fly ash, water glass modulus, and water–solid ratio on the properties of the material. Hu et al. [14] found that the compressive strength of AASF mortar increased with the increase in silicate modulus and alkali content, and decreased with the increase in fly ash content. Jin et al. [20] studied the formation law and polymerization reaction mechanism of polymerization products under different excitation conditions and found that the strong alkaline environment is the key factor for the dissolution of active substances and the formation of polymerization products. The combination effect of sodium hydroxide and sodium silicate solution is the best because they play a compatible coupling role. Through a series of experimental studies, Sun et al. [21] found that a higher w/b ratio can increase the workability and slightly reduce the strength, and a higher SiO_2_/Na_2_O ratio can improve the strength and workability. Yang et al. [22] studied the action mechanism of fly ash in slag–fly ash systems and found that the compressive strength increased first and then decreased with the decrease in fly ash content. At different ages, fly ash particles form embedded microstructures with different reaction degrees and sizes, which has an adverse impact on the mechanical properties of the material. The activation degree of fly ash gradually increases with the extension of age, which makes a continuous contribution to the later strength development. Samarakoon et al. [23] proposed two methods for introducing cement waste into partially alkaline cement binders (ACBs). The study finds glass powder-based activator to be 70% efficient in generating hydration products. The hydration product is dissolved in the binder phase, increasing the yield of the hydration product. Li et al. [24] replaced PC with a large amount of blast furnace slag (GGBFS) and fly ash (FA) to produce grouting materials with low cost, environmental friendliness, good workability, and excellent long-term performance. The study found that the incorporation of GGBFS (20%) + FA (50%) improved the fluidity, dispersibility, stability, and drying shrinkage properties of cement-based grouting.

According to the recent research results, the factors affecting the performance of alkali-activated slag–fly ash composite cementation systems are mainly concentrated in four aspects: fly ash content, water glass modulus, water glass solid content, and water–solid ratio. Scholars have studied the effects of some of these four factors on the properties of alkali-activated materials, but the effects of the changes of the four factors are rarely studied at the same time. There are few studies on the ranking of the effect of each parameter on the performance of the alkali-activated composite gelling system. In addition, there are few studies on the properties of alkali-activated slag materials with high fly ash content. Considering these aspects, this paper adopts the method of orthogonal experimental [25] design to adjust the content of fly ash, the modulus of water glass, the solid content of water glass, and the water–solid ratio. The effects of these four parameters on the fluidity, setting time, flexural strength, and compressive strength of the alkali-activated slag–fly ash composite cementitious system were studied. The microscopic analysis was carried out by means of a scanning electron microscope (SEM) and Fourier transform infrared spectroscopy (FTIR). Finally, it is found that each parameter affects the performance of the alkali-activated composite gelling system, the degree of influence is sorted, and the most important parameters affecting each performance are found.

## 2. Materials and Methods

### 2.1. Materials

#### 2.1.1. Slag

The test adopts s95 grade slag powder produced by Hebei Jinghang Mineral Products Co., Ltd., (Shijiazhuang, China). The inspection indicators are shown in Table 1. The inspected items meet the standard requirements of GBT 18046-2017 [26] for S95 grade slag powder.

#### 2.1.2. Fly Ash

The fly ash is Class I fly ash produced by Harbin Shuangda Fly Ash Products Co., Ltd., (Harbin, China). The inspection indicators are shown in Table 2. The inspected items meet the standard requirements of GBT 1596-2017 [27] for Class I fly ash.

#### 2.1.3. Alkaline Activator

The alkali activator is water glass, NaOH is the regulator of the modulus of water glass, and the water glass is liquid water glass produced by Foshan Kening New Material Technology Co., Ltd., (Foshan, China). The inspection indicators are shown in Table 3. The inspection items meet the requirements of GB/T 4209-2008 [28] standard. NaOH is a solid NaOH with a purity of 99% produced by Tianjin Tianyuan Chemical Co., Ltd., (Tianjin, China).

### 2.2. Test Preparation

#### 2.2.1. Matching Ratio Design

For single-factor or two-factor experiments, the design, implementation, and analysis of the experiments are relatively simple because of the small number of factors. However, in practical work, it is often necessary to examine three or more test factors at the same time. If a comprehensive test is carried out, the scale of the test will be very large, and it is often difficult to implement due to the limitation of test conditions. Orthogonal experimental design is a design method that uses orthogonal tables to arrange and analyze multi-factor experiments. It selects some representative level combinations from all the level combinations of the test factors to conduct the test and understands the overall test situation by analyzing the results of this part of the test.

The selection of the orthogonal test table should be based on the specific factors and levels of the test. In this test, four factors, such as fly ash content, water glass modulus, water glass solid content, and water–solid ratio, which affect the working performance and mechanical properties of the material, are selected. The activity of the slag is high, which has an important influence on the strength improvement of alkali-activated materials, but when the amount of slag is too high, it will have a negative impact on the working performance of alkali-activated materials, and even the phenomenon of “instant condensation” will occur. Fly ash has a positive effect on the working properties of the material, and the “ball bearing” effect of the fly ash particles can also improve the flow properties of the material [29]. At present, there are few studies on alkali-activated materials with high fly ash content, so four levels of 60%, 70%, 80%, and 90% with higher fly ash content are selected. For water glass, the water glass with higher modulus at room temperature has low solubility and low activity, and the water glass with low modulus has poor stability, is prone to coagulation, and the performance of the prepared samples is unstable [30], so if the modulus is too high or too low it will result in relatively low intensity [31]. Peng et al. showed that the performance of geopolymer materials is better when the modulus of water glass is 1.4 [32], so the modulus of water glass used in this experiment is four levels of 1.0, 1.2, 1.4, and 1.6. The solid content of water glass is the sum of the mass of sodium silicate and sodium oxide as a percentage of the total mass of water glass. The higher the solid content indicates the higher the alkali content involved in the alkali excitation reaction, and considering the economic factors, the solid content was finally determined to be 5%, 8%, 11%, and 14% at four levels. Han [33] studied the preparation of geopolymers with different water–binder ratios (0.29–0.41), and found that the unconfined compressive strength of the prepared geopolymers decreased with the increase in the water–binder ratio. The material with a water–binder ratio of 0.29 could not be formed, and the sample with a water–binder ratio of 0.32 had the best strength. In this paper, the water–solid ratio is set at four levels of 0.30, 0.33, 0.36, and 0.39 through the above research and pre-test. The factors and levels are shown in Table 4.

Using slag and fly ash as the main raw materials, water glass and NaOH as alkali activators, test groups with different mixing ratios were designed through orthogonal experiments, and the properties of the alkali-activated slag–fly ash composite system were tested and microscopically tested. The effects of fly ash content, water glass modulus, water glass solid content, and water–solid ratio on the material were studied. This test is a 4-factor and 4-level test. According to the orthogonal test rule, the L16 (4^5^) orthogonal test table with 5 factors and 4 levels is selected. The mix ratio design is shown in Table 5.

#### 2.2.2. Configuration of Alkaline Activator Solution

Because the water glass produced by different water glass manufacturers generally has different degrees of difference, the dosage of water glass cannot be determined very accurately. Therefore, this study selects the solid content of water glass as the parameter to control the dosage of water glass.

The initial modulus of water glass is 2.43, which is greater than the modulus of water glass required by the test. Therefore, the modulus of water glass needs to be adjusted lower. In this study, the modulus of water glass was adjusted by adding solid NaOH. According to the relative molecular mass of SiO_2_ and Na_2_O, the formula for calculating the modulus of water glass suitable for this experiment can be deduced. The process is as follows:

The relative molecular mass of SiO_2_ is 60.084.

The relative molecular mass of Na_2_O is 61.979.

The modulus of water glass is the ratio of the moles of silicon dioxide and sodium oxide in the composition of water glass, expressed in M, and calculated as follows:

(1) $$M=\frac{nSiO_{2}}{nNa_{2}O}$$

According to the relative molecular mass ratio of SiO_2_ and Na_2_O, the formula can be deduced as follows:

(2) $$M=\frac{m_{1}/60.084}{m_{2}/61.979}=\frac{m_{1}}{m_{2}}\times 1.0315$$

*m*_1_—the mass of *SiO*_2_;

*m*_2_—the mass of *Na*_2_*O*;

The initial modulus of water glass is 2.43. The following is an example of adjusting the target modulus of 100 g liquid water glass to 1.6:

The initial content of Na_2_O in 100 g liquid water glass is 13.73 g, and the initial content of SiO_2_ is 32.35 g. Assuming that the target modulus is 1.6, the content of NaO_2_ added to the water glass is *x*,

$$\begin{array}{l} 1.6=\;\frac{32.35}{x+13.73}\times 1.0315\\ \;x=7.13. \end{array}$$

That is, the Na_2_O that should be added is 7.13 g.

**Figure 1:**
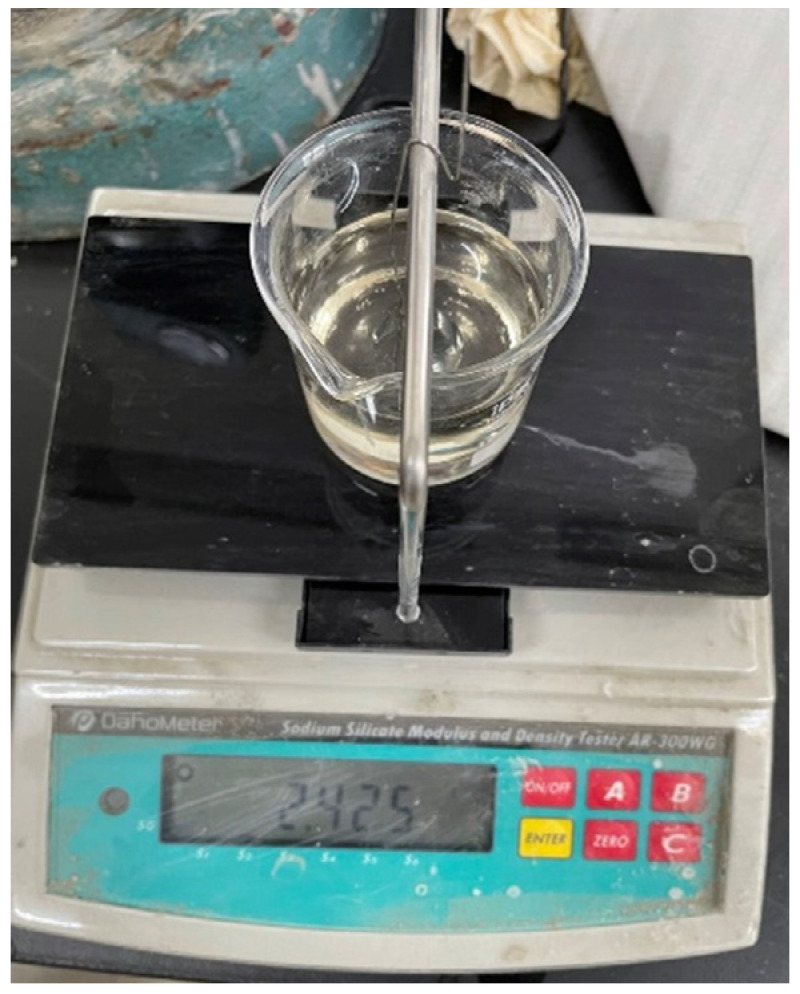
Test of water glass modulus.

Prepare water glass solution, and adjust the modulus of water glass by adding solid sodium hydroxide to the liquid water glass to make it reach the modulus required for the test. There will be exothermic problems during this process, so after adding sodium hydroxide and stirring it to dissolve, it needs to be sealed with plastic film and left for 24 h. The modulus is tested by a water glass modulus tester, as shown in Figure 1:

### 2.3. Test Preparation

#### 2.3.1. Fluidity Test

The fluidity of the material is based on the regulations in GBT 2419-2005 [34], but due to the large fluidity of the material, the size of the tabletop cannot meet the requirements for use, so the test method has been improved. Use a large smooth glass plate on a level table and wipe the glass plate and frustum mold with a damp cloth to slightly moisten the surface. Place the truncated cone die in the center of the glass plate, measure the diameters of the two vertical directions of the sample, and take the average value of the two as the fluidity of this group of specimens.

#### 2.3.2. Coagulation Time Test

The coagulation time refers to the provisions in GBT 1346-2011 [35] and is measured with a Vicat instrument. The initial and final coagulation times are obtained by measuring the time required for the test needle to sink into the depth.

#### 2.3.3. Flexural and Compressive Strength Testing

The strength refers to the provisions in GBT 17671-1999 [36], and the initial size of the specimen is a prism of 40 mm × 40 mm × 160 mm. The flexural strength and compressive strength of the 2 d, 7 d, and 28 d age specimens were tested using the YAW-300H mechanical testing machine produced by Jinan Hengruijin Testing Machine Co., Ltd., (Jinan, China). Among them, the compressive strength test needs to use a compressive fixture that meets the requirements of JC/T683 [37], and the compressive area is 40 mm × 40 mm fixture.

#### 2.3.4. SEM Observation

The microscopic morphology of the samples was observed by a new high-resolution field emission scanning electron microscope SUB80200 produced by HITACHI in Tokyo, Japan. After standard curing for 28 days, the prismatic block of 40 mm × 40 mm × 160 mm was crushed and sampled. The sample size was about 20 mm × 15 mm × 10 mm. Before scanning, the surface of the specimen should be sprayed with gold.

#### 2.3.5. FTIR Spectroscopy Test

The samples were prepared by the KBr tablet pressing method. Using a Therefisher Screen Nisolet iS 10 Fourier transform infrared spectrometer with a resolution of 4 cm^−1^, the number of scans is 32, and the test range is 400–4000 cm^−1^.

## 3. Results

### 3.1. The Effect of Each Parameter on the Flow Degree

**Figure 2:**
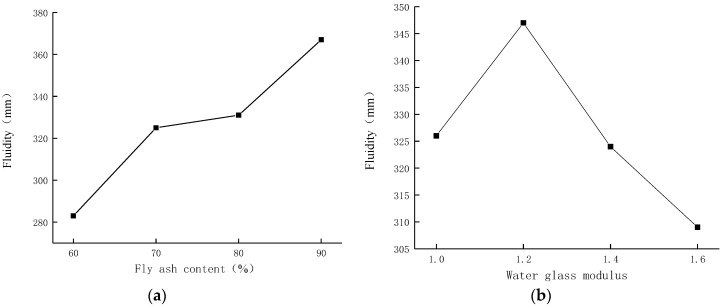

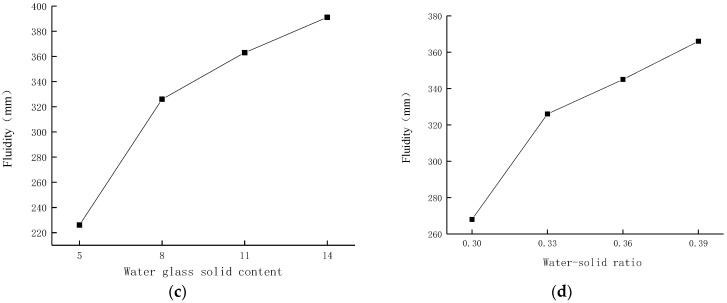
Influence of parameters on fluidity of composite gelling system. (**a**) Influence of fly ash content on fluidity. (**b**) Influence of water glass modulus on fluidity. (**c**) Influence of water glass solid content on fluidity. (**d**) Influence of water–solid ratio on fluidity.

The average results of the four groups with the same level of each factor are used for analysis. The fluidity test results of the alkali-activated slag/fly ash composite cementitious system are shown in Figure 2.

Figure 2a shows that as the amount of fly ash replacing slag increases from 60% to 90%, the fluidity of the composite gelling system increases gradually. It can be seen that the fluidity of the alkali-activated composite gelling system increases gradually with the increase in the fly ash content, which is due to the low alkali activity in the fly ash and the slow reaction with the alkali activator [18]. Figure 2b shows that when the modulus of water glass is in the range of 1.0–1.6, the fluidity first increases and then decreases with the increase in modulus. The more the content of water glass, the more viscosity will also increase, which will increase the fluidity of the material, but the modulus of water glass should not be too large, otherwise, the fluidity will decrease and affect its working performance. The modulus reaches the maximum value of 347 mm when the modulus is 1.2. Figure 2c,d show that the fluidity increases gradually with the increase in water glass solid content and water–solid ratio. Compared with the range of 8–14%, the fluidity of water glass increases faster in the range of 5–8%. The influence of the water–solid ratio on fluidity in the range of 0.30–0.33 is more obvious than that in 0.33–0.39. When the solid content of water glass is too low, the amount of SiO_2_ and Na_2_O participating in the reflection in the composite gelling system is insufficient, and some slag and fly ash fail to react, which affects the forming of the material and reduces the fluidity. The fluidity of the alkali-activated composite gelling system mainly comes from the adsorption of water on the surface of fly ash and slag particles to form a water film. With the increase in the water–solid ratio, the amount of mixing water increases, the adsorbed water on the particle surface increases, the water film becomes thicker, the lubrication between particles increases, and the fluidity increases.

The average values of the fluidity of fly ash content of 60%, 70%, 80%, and 90% are 282.500, 325.250, 330.500, and 366.500, respectively, and the range is R_A_ = 366.500 − 282.500 = 84.000. The average values of the fluidity of water glass modulus 1.0, 1.2, 1.4, 1.6 are 326.000, 346.500, 323.500, 308.750, and the range R_B_ = 346.500 − 308.750 = 37.75. The average values of the fluidity of water glass with solid content of 5%, 8%, 11%, and 14% are 225.500, 326.000, 362.750, and 390.500, respectively, and the range is R_C_ = 390.500 − 225.500 = 165.000. The mean values of fluidity with water–solid ratios of 0.30, 0.33, 0.36, and 0.39 are 268.250, 325.500, 344.750, and 366.250, respectively, and the range R_D_ = 366.250 − 268.250 = 98.000. Comparing the magnitudes of each R value, it can be seen that R_C_ > R_D_ > R_A_ > R_B_. According to the orthogonal design characteristics, the significant degree of the influence of the above four factors on the fluidity of the alkali-activated composite gelling system is ranked as follows: water glass solid content > water–solid content ratio > fly ash content > water glass modulus. It can be seen that adjusting the solid content of water glass is the primary factor to control the fluidity of the material. ANOVA was performed on the test results, and the F value determined the significance, with a larger F value indicating greater significance [38]. At the 5% significance level, the F values of fly ash content, water glass modulus, water glass solid content, and water–solid ratio were 4.530, 0.923, 19.904, and 6.766, respectively. The F value of the water glass solid content is significantly higher than that of the other three factors, which means that the water glass solid content has a greater effect on the fluidity than the other three parameters.

### 3.2. The Effect of Each Parameter on the Setting Time

**Figure 3:**
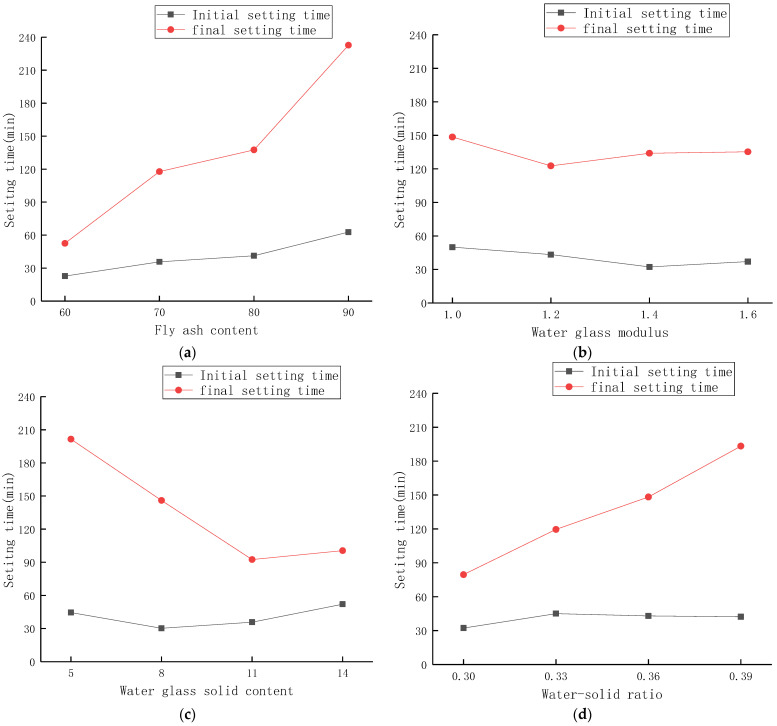
Influence of parameters on setting time of composite gelling system. (**a**) Influence of fly ash content on setting time. (**b**) Influence of water glass modulus on setting time. (**c**) Influence of water glass solid content on setting time. (**d**) Influence of water–solid ratio on setting time.

The average results of four groups with the same level of each factor were used for analysis. The setting time test results of the alkali-activated slag/fly ash composite cementitious system are shown in Figure 3.

It can be seen from Figure 3a that for the initial setting time, as the fly ash content changes from 60% to 90%, the initial setting time gradually becomes longer with the increase in the fly ash content. Further, it reaches the maximum when the fly ash content is 90%. Similarly, the effect of the amount of fly ash on the final setting time of the material is similar to that of the initial setting time, which gradually becomes longer with the increase in the fly ash content. This is because the calcium content of fly ash is much smaller than that of slag, so the setting time becomes longer with the increase in fly ash content. On the contrary, when the content of fly ash is low, the content of slag is high, and the content of Ca^2+^ in slag is much higher than that of fly ash. Free Ca^2+^ can make aluminosilicate raw materials form a number of condensation nodules at the beginning of depolymerization or geopolymerization, resulting in shortening the setting time. This confirms the theory that the setting time will be shortened with the increase in calcium ion content in the geopolymer [39,40]. In addition, as a charge balance ion, Ca^2+^ has strong electrostatic attraction and charge neutralization ability, which can promote the rapid formation of silicate polymers and shorten the setting time [41]. It can be seen from Figure 3b that for the initial setting time, as the water glass modulus changes from 1.0 to 1.6, the initial setting time first decreases and then increases with the increase in the water glass modulus. When the modulus is 1.4, the initial setting time is the shortest, which is 35.5% shorter than when the modulus of water glass is 1.0. The influence of the water glass modulus on the final setting time of the material is similar to that of the initial setting time, which first decreases and then increases with the increase in the water glass modulus. When the modulus is 1.2, the final setting time is the shortest, which is 17.3% shorter than that when the modulus of water glass is 1.0. As can be seen from Figure 3c, as the solid content of water glass changes from 5% to 14%, the initial setting time first decreases and then increases with the increase in the solid content of water glass. When the solid content is 8%, the initial setting time is the shortest, which is 41.8% shorter than that when the solid content of water glass is 14%. The effect of water glass solid content on the final setting time of the material is more obvious than that of the initial setting time. As the water glass solid content changes from 5% to 11%, the final setting time is shortened by 54.1%. This may be due to the fact that the concentration of OH^−^ ions increases with the increase in the solid content of water glass so that the glass bodies in fly ash and slag are continuously dissolved, resulting in a large amount of soluble Si and Al. With the occurrence of the dissolution-depolymerization process, there are SiO_4_^4−^ and AlO_4_^5−^ with different degrees of polymerization in the system, and the increase in the concentration of the activator base accelerates the formation of gels and shortens the final setting time. As can be seen from Figure 3d, as the water–solid ratio changes from 0.30 to 0.39, the initial setting time first increases and then decreases with the increase in the water–solid ratio. When the water–solid ratio is 0.33, the initial setting time is the longest, which is 39.5% longer than when the water glass solid content is 0.30. The influence of the water–solid ratio on the final setting time of the material is more obvious than that of the initial setting time. As the water–solid ratio changes from 0.30 to 0.39, the final setting time increases significantly. When the water–solid ratio is 0.39, the final setting time is 143.1% longer than that at 0.30. This is because, with the increase in water content, the molar concentration of the alkali activator is reduced, the speed of the geopolymerization reaction is slowed down, and the reaction time is prolonged.

According to the size of the range, the influence of various factors on the setting time of the alkali-activated slag–fly ash composite system can be determined. For the initial setting time, the range R_A_ of fly ash content is 40, the range R_B_ of water glass modulus is 17.75, the range R_C_ of water glass solid content is 21.75, and the range R_D_ of water–solid ratio is 12.75. Comparing the size of each R value, it can be seen that R_A_ > R_C_ > R_B_ > R_D_. According to the orthogonal design characteristics, the order of significance of the above four factors on the initial setting time of alkali-activated grouting materials is: fly ash content > water glass solid content > water glass modulus > water–solid ratio. The change of the content has the greatest influence on the initial setting time. Similarly, it can be seen that the final setting time is also the most affected by the amount of fly ash. ANOVA was performed on the final setting time. At the 5% significance level, the F values of fly ash content, water glass modulus, water glass solid content, and water–solid ratio were 10.181, 0.204, 4.606, and 4.209, respectively. The F value of fly ash content is significantly higher than that of the other three factors, which means that the effect of fly ash content on final setting time is greater than that of the other three parameters.

### 3.3. The Influence of Each Parameter on the Flexural Strength

**Figure 4:**
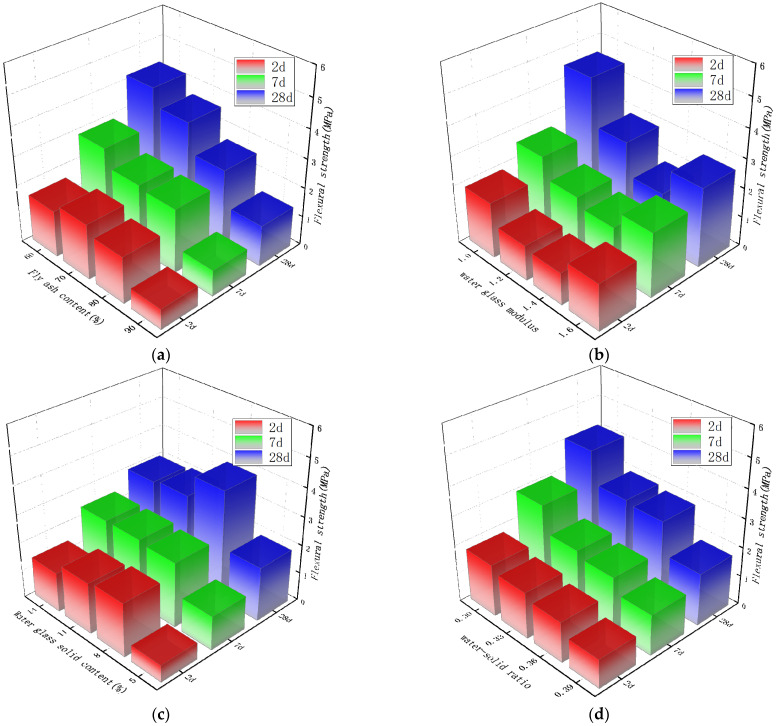
Influence of various parameters on the flexural strength of composite gelling system. (**a**) Influence of fly ash content on flexural strength. (**b**) Influence of water glass modulus on flexural strength. (**c**) Influence of water glass solid content on flexural strength. (**d**) Influence of water–solid ratio on flexural strength.

The average results of the four groups with the same level of each factor are used for analysis. The flexural strength test results of the alkali-activated slag/fly ash composite cementitious system are shown in Figure 4.

Slag and fly ash react under the action of an alkali activator to form C–S–H gel, C–A–S–H gel, and amorphous aluminosilicate gel. These gel products are the main source of strength formation of alkali-activated slag–fly ash composite cementation system. It can be seen from Figure 4a that with the increase in fly ash content, the flexural strength at 2 d age has a trend of increasing first and then decreasing. When the content of fly ash is 70%, the maximum strength is 1.9 MPa. For 7 d and 28 days, the flexural strength decreased with the increase in fly ash content. When the content of fly ash is 60%, the maximum is 2.9 MPa and 4.2 MPa, respectively, and the minimum is 0.9 MPa and 1.9 MPa when the content of fly ash is 90%. Figure 4b shows the influence of the modulus of water glass in the range of 1.0–1.6 on the flexural strength. The flexural strength of each age showed a trend of first decreasing and then increasing with the increase in the modulus of water glass, and it was the minimum when the modulus was 1.4. Figure 4c shows the effect of water glass solid content in the range of 5–14% on flexural strength. With the increase in solid content, the flexural strength at 2 d and 28 d age first increased and then decreased, and the flexural strength at 7 d age first increased and then stabilized at 2.4 MPa. Figure 4d shows the effect of different water–solid ratios on the flexural strength of the material. The influence of water–solid ratio on the flexural strength of each age is consistent, and the flexural strength of each age decreases with the increase in water–solid ratio.

According to the magnitude of the range, the influence of various factors on the flexural strength of the alkali-activated slag–fly ash composite system was judged. For the flexural strength at 2 d age, the range of fly ash content R_A_ is 1.275, the range of water glass modulus R_B_ is 0.775, the range of water glass solid content R_C_ is 1.300, and the range of water–solid ratio R_D_ is 0.800. Comparing the magnitude of each R value, it can be seen that R_C_ > R_A_ > R_D_ > R_B_. According to the orthogonal design characteristics, the significant degree of the influence of the above four factors on the early flexural strength of the alkali-activated composite cementitious system is as follows: water glass solid content > fly ash content > water–solid ratio > water glass modulus. This is because in the early stage of alkali-activated reaction, the amount of alkali will affect the reaction rate, and more C-S-H gel or C-A-S-H gel will be formed, which directly affects the early strength of the material [14]. Different from the 2 d age, the flexural strength of the material at 7 d and 28 d age was most affected by the content of fly ash. On the one hand, it is because, with the increase in age, a large amount of Ca^2+^ participates in the reaction to form the main solid phase that improves the strength of the material. The content of CaO in slag is much higher than that of fly ash, and the alkali activity is high, while the reactivity of fly ash under alkaline conditions is lower than that of slag. On the other hand, slag reactions mainly form C–A–S–H gels, while fly ash usually forms amorphous aluminosilicate gels. The change in fly ash content causes the change in the number of different types of reaction products, which in turn affects the change in strength. ANOVA was performed on the flexural strength at 2 d age. At the 5% significance level, the F values of fly ash content, water glass modulus, water glass solid content, and water–solid ratio were 13.167, 6.204, 15.352, and 5.056, respectively. The F values of fly ash content and water glass solid content were significantly higher than those of the other two factors. This means that the fly ash content and water glass solid content has a greater impact on the early flexural strength than the other two parameters.

### 3.4. The Influence of Each Parameter on the Compressive Strength

**Figure 5:**
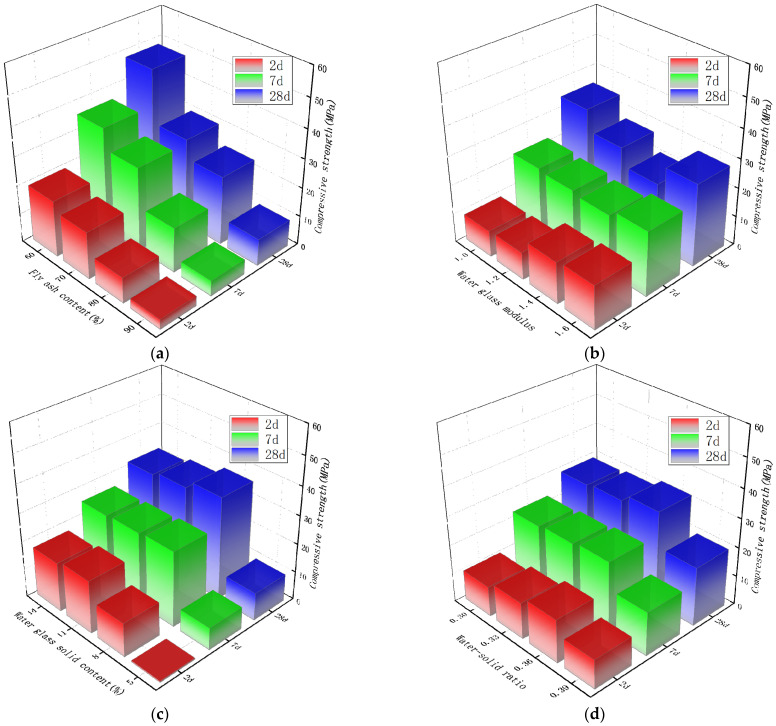
Influence of various parameters on the compressive strength of composite gelling system. (**a**) Influence of fly ash content. (**b**) Influence of water glass modulus on compressive strength on compressive strength. (**c**) Influence of water glass solid content. (**d**) Influence of water–solid ratio on compressive strength on compressive strength.

The average results of the four groups with the same level of each factor are used for analysis. The compressive strength test results of the alkali-activated slag/fly ash composite cementitious system are shown in Figure 5.

Figure 5a shows the effect of different fly ash content on the compressive strength of the material. As the fly ash content increases from 60% to 90%, the compressive strength at each age gradually decreases. When the content of fly ash is 60%, the compressive strength is the maximum value. The maximum compressive strength of 2 d, 7 d, and 28 d are 19.85 MPa, 35.9 MPa, and 48.2 MPa, respectively. Figure 5b shows the influence of the water glass modulus on the compressive strength in the range of 1.0–1.4. With the increase in the modulus of water glass, the compressive strength of the material at 2 d age showed an increasing trend, and the maximum value was 15.2 MPa. The compressive strength at the age of 7 d did not change significantly and was basically stable at 21 MPa. The compressive strength at the age of 28 d showed a trend of first increase and then decrease, and the minimum value was 21.3 MPa when the modulus was 1.4. An increase in the modulus of water glass will lead to the formation of C-(A)-S-H with a lower Ca/Si ratio and molar volume. It has a higher surface area, which contributes to the increase in strength. It should be noted that when the modulus is too high, the alkalinity inside the sample may decrease, which may lead to a decrease in compressive strength. Figure 5c shows the influence of water glass solid content in the range of 5–14% on compressive strength. With the increase in solid content, the compressive strength of each age shows an increase first and then a decreasing trend. Among them, the compressive strength of the 2 d age reaches the maximum value of 18.6 MPa when the solid content is 11%, and the 7 d and 28 d age reaches the maximum value of 26.5 MPa and 36.2 MPa when the solid content is 8%. In general, as the solid content of water glass increases, more C-S-H gel or C-A-S-H gel is formed, and the increase in solid content will accelerate the reaction of slag and fly ash. However, when the solid content exceeds 8%, the compressive strength decreases with the increase in the solid content, indicating that the compressive strength of the material has an optimal water glass solid content. Figure 5d shows the effect of different water–solid ratios on the compressive strength of the material. With the increase in water–solid ratio, the changing trend of compressive strength at each age is basically the same, showing a trend of first increase and then decrease, reaching the maximum value at a water–solid ratio of 0.36, which are 15 MPa, 24.4 MPa, and 32.3 Mpa, respectively.

According to the magnitude of the range, the influence of various factors on the flexural strength of the alkali-activated slag–fly ash composite system was judged. Similar to the flexural strength, the water glass solid content has the greatest influence on the compressive strength of the material at the early 2 d age, and the compressive strength of the material at the 7 d and 28 d age are most affected by the content of fly ash. It can be seen that controlling the solid content of water glass is the primary factor to adjust the early strength of the material. If you want to adjust the mid- and late-stage strength of the material, controlling the amount of fly ash is the primary factor. ANOVA was performed on the compressive strength at 2 d age. At the 5% significance level, the F values of fly ash content, water glass modulus, water glass solid content, and water–solid ratio were 10.688, 2.085, 13.919, and 1.062, respectively. The F values of fly ash content and water glass solid content were significantly higher than those of the other two factors. This means that compared with the other two parameters, the content of fly ash and the solid content of water glass have a greater influence on the early compressive strength of the material. This is consistent with flexural strength.

### 3.5. SEM Image Analysis

**Figure 6:**
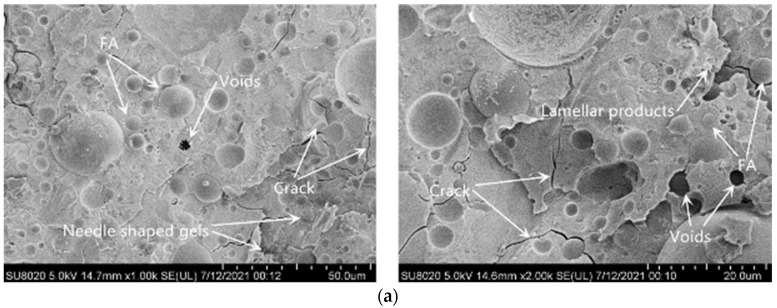

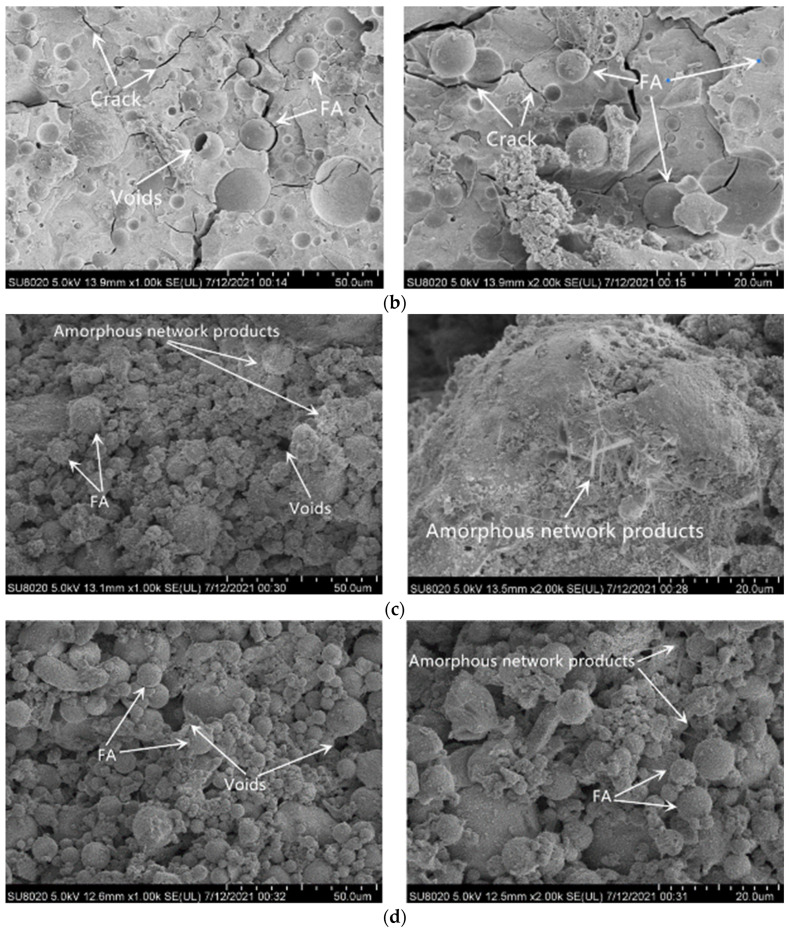
SEM image of 28 d specimen. (**a**) Group 2 samples, (**b**) Group 3 samples, (**c**) Group 15 samples, (**d**) Group 16 samples.

The internal microstructure of the material plays a very important role in the macroscopic mechanical properties of the material. In this study, groups 2 and 3 with higher compressive strength at 28 days and groups 15 and 16 with lower compressive strength were selected for the SEM test. Figure 6a–d show the microscopic morphologies of reaction products in groups 2, 3, 15, and 16, respectively.

Figure 6a,b show that under the action of the alkali activator, the gel products generated by the reaction of groups 2 and 3 make the overall microstructure of the samples relatively dense. The reaction produces needle-like and layered gel products. No obvious slag particles can be seen in the figure, most of which have participated in the reaction, and a small number of fly ash particles (FA) have not reacted completely or maintained their original form. These fly ash particles with different particle sizes and degrees of reaction, as well as the remaining shell after the reaction, are intercalated between the reaction-generated amorphous gels to form a monolithic structure, similar to that found by Chi [25]. Due to the chemical shrinkage and fine pore structure of the material, the internal relative humidity drops sharply, resulting in large pore pressure. Pore pressure induces not only elastic deformation of the slurry but also large creep of the slurry [42], leading to the generation of shrinkage cracks in Figure 6a,b. Figure 6c,d show that the overall performance of the microscopic morphology in groups 15 and 16 is significantly less dense than that in groups 2 and 3. The gel products generated by the reaction are few and cannot sufficiently fill the pores between the particles. Most of the fly ash particles in the matrix did not fully participate in the reaction, there was no effective connection between the particles, the overall appearance was relatively loose, and irregular network products were seen in the reaction products. Comparing Figure 6a–d, it can be seen that the gel product produced by the alkali-activated composite gelling system under the condition of increasing slag and alkali activator will also increase, resulting in a very structurally dense material, and the strength of the material will also increase. On the contrary, the content of fly ash instead of slag increases, the specific surface area of fly ash is too large, the liquid phase environment of alkali-activated reaction is insufficient, the reaction of fly ash particles is insufficient, and the generated gel product decreases accordingly. As a result, the overall microstructure is relatively loose, and the strength of the material will also decrease [43].

### 3.6. FTIR Analysis

**Figure 7:**
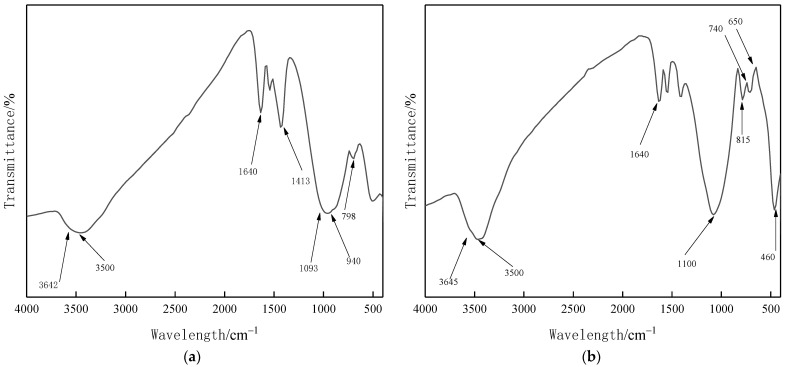
FTIR plots of slag and fly ash. (**a**) Slag, (**b**) Fly ash.

**Figure 8:**
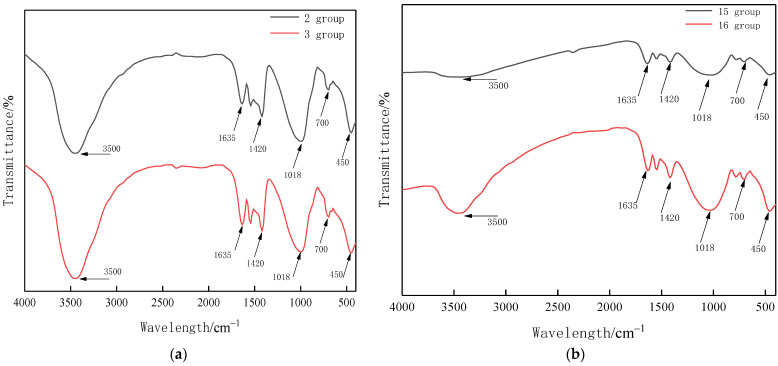
FTIR images of the 2nd, 3rd, and 15th, 16th groups of the composite gelling system. (**a**) 2 and 3 groups, (**b**) 15 and 16 groups.

The samples were prepared by the KBr tablet method, and a Therefisher Screen Nisolet IS 10 Fourier transform infrared spectrometer was used with a resolution of 4 cm^−1^, 32 scans, and a test range of 400–4000 cm^−1^. The raw materials of slag and fly ash, as well as groups 2 and 3 with higher compressive strength of 28 d and groups 15 and 16 with lower compressive strength were tested, and the results are shown in Figure 7 and Figure 8.

Figure 7a is the FTIR image of the slag. The absorption peak at 1093 cm^−1^ is caused by the antisymmetric stretching vibration of Si–O–Si. The Si–O bond symmetrical stretching vibration and bending vibration cause the absorption peak at 798 cm^−1^. The absorption peak at 940 cm^−1^ is caused by the bending vibration of Si-OH. Among them, the -OH antisymmetric stretching vibration and H–O–H bending vibration of structural water cause absorption peaks at 3500 cm^−1^ and 1640 cm^−1^. Figure 7b is the FTIR image of fly ash. The absorption peak at 3500 cm^−1^ is caused by –OH stretching vibration and H-O-H bending vibration. The absorption peak at 1100 cm^−1^ is caused by the stretching vibration of Al–O and Si–O. Al–O and Si–O symmetrical stretching vibrations lead to absorption peaks at 650 cm^−1^ and 740 cm^−1^. The absorption peak at 460 cm^−1^ is caused by vibrational bending in the Si–O bond plane.

It can be seen from Figure 8 that the absorption peak at 3500 cm^−1^ is caused by the stretching vibration of hydroxyl groups in Ca–OH_2_. The bending vibration of H–O–H induces an absorption peak at 1635 cm^−1^. The absorption peak at 1420 cm^−1^ is due to the asymmetric stretching vibration and out-of-plane bending vibration of the O–C–O bond in CO_3_^2−^. The absorption peak at 1018 cm^−1^ is caused by the V_3_ vibration of SiO_4_^2−^. 700 cm^−1^ is caused by the V_4_ vibration of SiO_4_^2−^. 450 cm^−1^ is caused by the V_2_ vibration of SiO_4_^2−^. The absorption peaks corresponding to silicate ions are 1018 cm^−1^, 700 cm^−1,^ and 450 cm^−1^, the peak areas of 2 and 3 groups are 383.34 and 374.43, and the peak areas of 15 and 16 groups are 487.87 and 454.2. Due to the low strength of groups 15 and 16, it is directly proved from the microscopic point of view that the content of silica can improve the macroscopic mechanical properties of the material. However, when the amount of silica is too high, the coagulation time of the material is too early, resulting in insufficient internal molecular reaction, resulting in a phenomenon of low macroscopic mechanical properties.

## 4. Conclusions

In this paper, the solution of liquid water glass and solid sodium hydroxide is used as the alkaline activator, and the orthogonal test method is used to design the mix ratio. The effects of four parameters of fly ash content, water glass modulus, water glass solid content, and water–solid ratio on the working performance and mechanical properties of the alkali-activated slag–fly ash composite cementation system were discussed. And the materials were studied by microscopic tests such as SEM and FTIR. The following conclusions can be drawn from the test results:

(1) The order of significance of the influence of the four parameters on the fluidity of the material is: water glass solid content > water–solid ratio > fly ash content > water glass modulus. That is, the change in the solid content of water glass has the greatest influence on fluidity. The amount of water glass solid content determines whether the amount of SiO_2_ and Na_2_O involved in the alkali-activated reaction is sufficient. Controlling the water glass solid content is the key to ensuring the fluidity of the material.
(2) The change in fly ash content had the greatest influence on the setting time, and the setting time gradually became longer with the increase in fly ash content. The content of fly ash affects the content of Ca^2+^ in the material, which in turn affects the formation of multiple condensation nuclei in the initial process of depolymerization of silico-alumina raw materials. This eventually leads to a change in setting time. A key control for adjusting the setting time of the material is the fly ash content.
(3) In the early stage of an alkali-activated reaction, the amount of alkali will affect the reaction rate. More C-S-H gel or C-A-S-H gel is formed, which directly affects the early strength of the material. Controlling the solid content of water glass is the primary factor for adjusting the early strength of the material. With the growth of age, a large amount of Ca^2+^ participates in the reaction to form the main solid phase that improves the strength of the material. The mechanical strength of the material at 7 d and 28 d age are most affected by the content of fly ash. Controlling the solid content of water glass is the primary factor for adjusting the mid- and late-stage strength of the material.The resulting gel product increases with the addition of slag and alkali activator, resulting in a very dense structure and increased strength of the material. When the amount of silica in the material is too high, the phenomenon of lower macroscopic mechanical properties occurs.

In this paper, the solution of liquid water glass and solid sodium hydroxide is used as the alkaline activator, and the mixing ratio is designed by the orthogonal test method. The influence law of four parameters of fly ash content, water glass modulus, water glass solid content, and water–solid ratio on the working performance and mechanical properties of alkali-activated slag–fly ash composite system was discussed, and the degree of impact was sorted to find the most important parameters affecting each performance. The gelling system was analyzed by microscopic tests such as SEM and FTIR. Alkali-activated materials, as an alternative to cement, are environmentally friendly materials with great potential for development. The effects of various parameters on the durability properties of the alkali-activated slag–fly ash composite systems, such as impermeability, frost resistance, and shrinkage, are worthy of further study.

## Figures and Tables

**Table 1:** S95 slag inspection index.

Test Items	Unit	Standard Indicator	Test Result
Density	g/cm^3^	≥2.8	2.8
Specific surface area	m^2^/kg	≥350	350
7 d activity index	%	≥75	76
28 d activity index	%	≥95	96
Mobility ratio	%	≥90	90
Moisture content (mass fraction)	%	≤1.0	1.0
Sulfur trioxide (mass fraction)	%	≤4.0	4.0
Chloride (mass fraction)	%	≤0.06	0.02
Loss on ignition (mass fraction)	%	≤3.0	3.0

**Table 2:** Fly ash inspection index.

Test Items	Unit	Standard Indicator	Test Result
Fineness	%	≤12.0	10.1
Water demand	%	≤95	92
Loss on ignition	%	≤5.0	2.2
Water content	%	≤1.0	0.1
Density	g/cm^3^	≤3.0	2.3
Sulfur trioxide	%	≤4.0	1.2
Intensity activity index	%	≥70.0	75.9

**Table 3:** Liquid water glass inspection index.

Test Items	Unit	Standard Indicator	Test Result
Exterior	--	Liquid sodium silicate is a slightly colored translucent viscous liquid	Slightly colored translucent viscous liquid
Baume degree (20 °C)	Be	49–51	50
Iron (Fe) content	%	≤0.02	0.01
Water-insoluble content	%	≤0.20	0.06
Density (20 °C)	g/cm^3^	1.52–1.59	1.53
Sodium oxide (Na_2_O) content	%	≥12.8	13.73
Silicon dioxide (SiO_2_) content	%	≥29.2	32.35
Modulus (M)	--	2.2–2.5	2.43

**Table 4:** Orthogonal factor level table.

Level	(A) Fly Ash Content	(B) Water Glass Modulus	(C) Water Glass Solid Content	(D) Water–Solid Ratio
1	60%	1.0	5%	0.30
2	70%	1.2	8%	0.33
3	80%	1.4	11%	0.36
4	90%	1.6	14%	0.39

**Table 5:** Test grouping.

Level	(A) Fly Ash Content	(B) Water Glass Modulus	(C) Water Glass Solid Content	(D) Water–Solid Ratio
1	60%	1.0	5%	0.30
2	60%	1.2	8%	0.33
3	60%	1.4	11%	0.36
4	60%	1.6	14%	0.39
5	70%	1.0	8%	0.36
6	70%	1.2	5%	0.39
7	70%	1.4	14%	0.30
8	70%	1.6	11%	0.33
9	80%	1.0	11%	0.39
10	80%	1.2	14%	0.36
11	80%	1.4	5%	0.33
12	80%	1.6	8%	0.30
13	90%	1.0	14%	0.33
14	90%	1.2	11%	0.30
15	90%	1.4	8%	0.39
16	90%	1.6	5%	0.36

## Data Availability

No new data were created or analyzed in this study. Data sharing is not applicable to this article.
